# Lysozyme: an endogenous antimicrobial protein with potent activity against extracellular, but not intracellular *Mycobacterium tuberculosis*

**DOI:** 10.1007/s00430-024-00793-0

**Published:** 2024-06-20

**Authors:** Felix Immanuel Maier, David Klinger, Mark Grieshober, Reiner Noschka, Armando Rodriguez, Sebastian Wiese, Wolf-Georg Forssmann, Ludger Ständker, Steffen Stenger

**Affiliations:** 1 Institute of Medical Microbiology and Infection and Hygiene, Ulm University, Ulm, Germany; 2 Core Facility of Functional Peptidomics, Ulm University, Meyerhoferstraße 4, 89081 Ulm, Germany; 3 Core Unit Mass Spectrometry and Proteomics, Ulm University, Albert Einstein Allee 23, 89081 Ulm, Germany; 4 Pharis Biotec GmbH, Feodor-Lynen. Str. 31, 30625 Hannover, Germany

**Keywords:** Tuberculosis, Antimicrobial peptides, Human, Macrophages, Lysozyme

## Abstract

Endogenous antimicrobial peptides (AMPs) play a key role in the host defense against pathogens. AMPs attack pathogens preferentially at the site of entry to prevent invasive infection. *Mycobacterium tuberculosis* (*Mtb*) enters its host via the airways. AMPs released into the airways are therefore likely candidates to contribute to the clearance of *Mtb* immediately after infection. Since lysozyme is detectable in airway secretions, we evaluated its antimicrobial activity against *Mtb*. We demonstrate that lysozyme inhibits the growth of extracellular *Mtb*, including isoniazid-resistant strains. Lysozyme also inhibited the growth of non-tuberculous mycobacteria. Even though lysozyme entered *Mtb*-infected human macrophages and co-localized with the pathogen we did not observe antimicrobial activity. This observation was unlikely related to the large size of lysozyme (14.74 kDa) because a smaller lysozyme-derived peptide also co-localized with *Mtb* without affecting the viability. To evaluate whether the activity of lysozyme against extracellular *Mtb* could be relevant in vivo, we incubated *Mtb* with fractions of human serum and screened for antimicrobial activity. After several rounds of sub-fractionation, we identified a highly active fraction-component as lysozyme by mass spectrometry. In summary, our results identify lysozyme as an antimycobacterial protein that is detectable as an active compound in human serum. Our results demonstrate that the activity of AMPs against extracellular bacilli does not predict efficacy against intracellular pathogens despite co-localization within the macrophage. Ongoing experiments are designed to unravel peptide modifications that occur in the intracellular space and interfere with the deleterious activity of lysozyme in the extracellular environment.

## Introduction

Infections with drug-resistant pathogens are an increasing challenge worldwide. The motivation for global pharmaceutical companies to intensify efforts to develop new antimicrobials is limited. This is due to the immense efforts required for conducting large clinical studies and aggravated by the limited financial profitability of anti-infective treatment. This is particularly problematic for diseases with a high prevalence in low-income countries such as tuberculosis. Each year over 10 million individuals develop active tuberculosis of which more than 1.2 million die. The outcome of an infection with *Mycobacterium tuberculosis* (*Mtb*) is dependent on host- and pathogen factors. Besides the strain-specific virulence (quote), drug-resistance of *Mtb* is a major obstacle for the successful treatment of tuberculosis. The proportion of infections with resistant strains in 2021 was 3.4% [51]. Despite the successful implementation of Delamanid, Bedaquilin and most recently Pretomanid there is desperate need for efficient and tolerable antituberculosis drugs ideally with distinct mechanisms of action.

Antimicrobial proteins and peptides (AMPs) have the potential to complement and improve conventional antibiotic therapy. AMPs inhibit a broad spectrum of microorganisms such as bacteria, fungi, viruses or parasites [17, 26, 39]. AMPs are produced by microorganisms themselves, plants, animals or endogenously by human cells. Here, AMPs are detected in tissues, surface epithelia, fluids and inside immune cells such as leukocytes or macrophages [17, 18, 35, 41, 42]. In principle, AMPs act by lysis of bacterial cell walls [4, 53] or penetration into the bacteria to inhibit protein synthesis or enzymatic activity [5]. Exposure to AMPs rarely induces the development of drug resistance [4, 16].

However, natural resistance should be ruled out before AMPs are used. Bacteria have developed various mechanisms to reduce the activity of AMPs. They have modified the structure and composition of their cell wall [2, 44] or secrete bacterial proteases [4, 53].

Vor ihrem Einsatz sollten jedoch natürliche Resistenzen ausgeschlossen werden. Bakterien haben verschiedene Mechanismen entwickelt, um die Aktivität von AMPs zu verringern. Sie haben beispielsweise Struktur und Zusammensetzung ihrer Zellwand modifiziert [6,93] oder sezernieren bakterielle Proteasen [11,112].

Several endogenous AMPs, such as lactoferrin [46], human β−Defensin [11], LL-37 [39], granulysin [47] and lysozyme [22, 25] have been associated with protection against tuberculosis. Human lysozyme belongs to a group of enzymes first described by Alexander Fleming in 1922 [13, 54]. Lysozymes can be categorized in three subgroups: c-Lysozyme (chicken/conventional), g-Lysozyme (goose) and i-Lysozyme (invertebrate). Human lysozyme belongs to the c-Lysozymes [7] and consists of 14.4 kDa and 130 amino acids [1, 7, 20, 23, 24]. Lysozyme occurs in several compartments of the human body, such as serum, urine, cerebrospinal fluid, airways, gastrointestinal tract, breast milk and saliva [6, 8, 10, 15]. Mainly, lysozyme is produced by macrophages and neutrophils, where the protein is stored in intracellular granules [7]. Production and secretion of lysozyme is regulated by bacterial lipopolysaccharide and cytokines, including IFN-γ or TNF-α [27]. Lysozymes hydrolyze the β-1,4-glycosidic bindings between the bacterial N-Acetylglucosamine and the N-Acetyl-muraminacid of the peptidoglycans of the bacterial cell wall, which causes cell lysis [7, 9, 49]. In addition, c-Lysozymes induce pores in bacterial cell walls [38, 56]. However, a possible bacterial resistance to lysozyme has to be discovered before its clinical use (as mentioned above) as modulations of the bacterial cell wall may affect these mechanims of acting of lysozyme.

Due to the small size, broad antimicrobial activity, the unique mechanism of action and the favorable tolerability in vivo, lysozyme is an attractive candidate for complementing the conventional treatment of severe infectious diseases. The objective of this study was to investigate the interaction of human lysozyme with the prototypic intracellular pathogen *Mtb*.

## Materials and methods

### Source and culture of mycobacteria

All used mycobacterial strains are shown in Table 1. Mycobacteria were amplified, stored and cultured as described previously [33]. Representative vials were thawed and enumerated for viable CFU on Middlebrook 7H11 plates (BD Biosciences). Live-dead staining (BacLight, Invitrogen) of bacterial suspensions with fluorochromic substrates revealed a viability of the bacteria > 90%. rior to use, aliquots were sonicated in a water bath for 10 min at 40 kHz and 110 W at room temperature to disrupt small aggregates of bacteria. The cell culture of mycobacteria occurred on Middlebrook 7H11 agar plates. 2 × 10^6^ sonicated mycobacteria were disseminated on the plates in variant logarithmic dilutions (1:10, 1:100 and 1:1000). Then an incubation occurred for 14 days at 37 °C. After the incubation, the colonies were CFU.

**Table 1:** Mycobacterial strains

Name	Source
*Mycobacterium tuberculosis*	ATCC 27,294^a^
*Mycobacterium kansasii*	ATCC 12,478^a^
*Mycobacterium fortuitum*	ATCC 6841^a^

^a^_ATCC (American Type Culture Collection) Manassas, VA, USA_

### ^3^ H-Uracil proliferation assay

As a correlate of mycobacterial viability, we measured the incorporation of radioactively-labelled 5.6-^3^H-uracil (ART-0282, Biotrend, Cologne, Germany) into the bacterial RNA [33]. 2 × 10^6^ sonicated mycobacteria were incubated with the peptides in Middlebrook 7H9 broth in a 96-well plate. All samples were set up in triplicate, using 2 µg/ml rifampin (*Mtb*) or 2 µg/ml clarithromycin (NTM) as controls. ^3^H-Uracil (0.3 µCi/well) was added after 72 h for *Mtb* or 48 h for NTM and cultures were incubated for an additional 18 h. Mycobacteria were then inactivated by treatment with 4% paraformaldehyde (PFA, Sigma-Aldrich) for 30 min and transferred onto glass fiber filters (Printed Filtermat A, PerkinElmer) using a 96-well based Filtermat Harvester (Inotech). Fiber filters were dried in a microwave at 240 W for 5 min and sealed at 75 °C with a sheet of solid scintillant wax (MeltiLex, PerkinElmer). Radioactivity was measured using a β-Counter (Sense Beta, Hidex). Antimicrobial activity (%) was calculated as counts per minute (cpm) of the treated sample/cpm of the un-treated sample × 100.

### Infection of macrophages with Mtb

As described previously, human peripheral blood mononuclear cells (PBMC) were isolated from buffy coats of anonymous donors (Institute of Transfusion Medicine, Ulm University) by density gradient centrifugation (Ficoll-Paque Plus, GE Healthcare). Monocytes were selected from plastic adherence and thoroughly washed. For generation of monocyte-derived macrophages, cells were cultured in M-SFM with granulocyte–macrophage colony-stimulating factor (GM-CSF, 10 ng/ml, Miltenyi) for 6 days as described previously [33]. Afterwards, macrophages were detached using EDTA (1mM, Sigma-Aldrich) and bulk-infected in 6-well plates with single-cell suspensions of *Mtb* at a multiplicity of infection of 5. After 2 h, cells were thoroughly washed to remove extracellular bacteria and harvested using 1 mM EDTA (Sigma-Aldrich). The infection rate was regularly determined by auramine-rhodamine staining and showed a donor-dependent variability between 17 and 32%. The number of bacilli per infected macrophages was within the range of 1–3.

### Quantification of intracellular mycobacterial growth: mycobacterial growth inhibition assay (MGIA)

Macrophages were bulk-infected with *Mtb* (MOI = 5, 2 h) and extracellular bacilli were removed by thorough rinsing, harvested and seeded in a 24-well plate at a concentration of 1 × 10^5^ cells in 300 µl M-SFM. Infected macrophages were treated as indicated and after 72 h cells were lysed by adding 200 µl of water. The lysates were transferred to mycobacteria growth indicator tubes (MGIT, BD Biosciences, Franklin Lakes, USA), supplemented with 800 µl supplement containing oleic acid, albumin, dextrose and catalase (OADC, BD Biosciences) and incubated in a Bactec MGIT 320 system (BD Biosciences). The Bactec MGIT 320 detects oxygen consumption and gives the exact time (in minutes) from the beginning of culture to the detection of bacterial activity (time to positivity). The number of viable bacilli was calculated by comparison of the time to positivity in the sample with a standard curve obtained from the growth of tenfold diluted mycobacterial suspensions (10^3^ to 10^7^ bacilli, duplicate).

### Peptide origin and synthesis

Lysozyme was extracted and purified from human neutrophil granulocytes (Sigma-Aldrich). Lys-H1 (KVFERCELARTLKRLGM) was synthesized by PSL Heidelberg (Heidelberg, Germany) using F-moc chemistry [33]. For visualization of Lys-H1, the peptide was conjugated N-terminally to the fluorescent dye Atto647N (PSL Heidelberg). Peptides were purified to > 95% homogeneity by reverse-phase HPLC. To allow a valid comparison between the experiments, all concentrations are given in molarity. This partially resulted in uneven concentrations and non-linear titration steps.

### Detection of endogenous lysozyme in macrophages

*Mtb*-infected macrophages were distributed on 8-chamber slides (Thermo Fisher Scientific; final volume 100 µl). After fixation of the cells with PFA (Sigma; final concentration 4%), non-specific binding sites are blocked using a blocking buffer (1 h at room temperature). This additionally permeabilizes the cell wall for intracellular staining. The antibodies are diluted and pipetted one after the other in a dark chamber (1 h incubation each). After staining, the slides were examined under the microscope for lysozyme, *Mtb*, and a colocalization of both.

All used antibodies are shown in Table 2. Quantification of colocalization was determined by scanning 10 fields of view per experiment with the software ZEN (Zeiss) and ImageJ (Wayne Rasband).

**Table 2:** Antibodies

Antibody	Source
α human Lysozyme unconjugated	Abcam
α Lipoarabinomannan (LAM) unconjugated	CS-35
donkey α rabbit Alexa647	Dianova
donkey α rabbit Cy2	Dianova
goat α mouse Cy3	Dianova

### Uptake of Lys-H1 in macrophages

To assess the uptake of Lys-H1 in macrophages, the peptide was labeled with the fluorescent Atto647N by PSL Heidelberg. 1 × 10^5^ macrophages were incubated with 15 µM Lys-H1-Atto in an 8-chamber-slide, unstimulated macrophages were used as negative control. After 24 h, the supernatant was removed and cells were fixed using 4% paraformaldehyde (PFA), followed by blocking in 0,5% bovine serum albumin (BSA). Cell nuclei were stained with DAPI (5µg/ml). Representative pictures of five different donors were acquired using the inverted laser scanning confocal microscope LSM 710 (Zeiss, Oberkochen, Germany). Image processing was performed using ImageJ software (v 1.52c). All images displayed in this study were processed for brightness/contrast. *Mtb* were resuspended in 500 µl dye buffer and washed twice at 10.000 rpm for 10 min. The pellet was resuspended in 50 µl dye buffer and 5 µl Succinimidyl-Ester-488 (invitrogen, Waltham, USA) was added for one hour. In a 24-well plate, about 5 × 10^5^ macrophages were incubated with the succinimidyl-ester stained *Mtb* for 2 h, using a multiplicity of infection of 12,5. Afterwards, 50 nM Lys-H1-Atto (lower concentration required due to higher sensitivity compared to microscopy) were added for 24 h. The supernatant was transferred into round bottom tubes and the remaining macrophages were removed from the wells using 1mM PBS-EDTA (Thermo Fisher Scientific) and transferred into the corresponding tubes. The uptake of *Mtb* and Lys-H1-Atto in macrophages was measured using the flow cytometer FACS Canto II (Becton Dickinson, Franklin Lakes, USA) with FlowJo. For investigation of Lys-H1 cell-specific uptake, macrophages were seeded in sterile FACS tubes alongside autologous PBMC in a 1:1 ratio for a total cell count of 0.5 × 10^6^ cells in AIM V medium [33]. Cells were incubated with Lys-H1-Atto647N for 2 h at 37 °C. Afterwards, cells were washed with FACS buffer and centrifuged for 10 min at 1300 rpm. Supernatant was discarded and cells were stained against MHC class II by a FITC-conjugated HLA-DR antibody (1:200). Sample analysis was performed using a FACSCalibur flow cytometer (BD Biosciences). Data analysis was performed using FlowJo Version 10.5.3 (BD Biosciences) and GraphPad Prism Version 8.2.1 (GraphPad Software).

### Screening of haemofiltrates

Haemofiltrates of unknown concentration were dissolved in aqua. Starting from thousands of litres of haemofiltrate, a waste product of haemodialysis of patients with renal failure, a peptide library was generated [43]. Peptides were separated into 300–500 pools based on charge (cation-exchange separation) and hydrophobicity (reversed-phase liquid chromatography). This haemofiltrate peptide library is a salt-free source of highly concentrated peptides and small proteins (< 30 kDa) which can be exploited for the unbiased search for antimicrobial peptides (Bosso 2018). Subsequently, an ^3^H-Uracil proliferation assay was performed with the addition of different haemofiltrates fractions as described above. Two additional rounds of chromatographic purifications were performed to obtain a pure active compound. The active chromatographic fraction from the peptide bank was subjected to reversed-phase chromatography on Source RPC 15 (Cytiva, USA) of dimensions 2 × 25 cm. at a flow rate of 13 ml/min, using the gradient of acetonitrile 0/5, 10/25, 15/30/ 55/50, 65/60 and 75/100 (tun time, min/%B) The second purification step was performed on a Phenomenex Aqua RP18 (Phenomenex, USA) of dimensions 10 × 25 cm at a flow rate of 2.5 mL/min, using the gradient of acetonitrile 0/5, 10/30, 15/35, 55/45, 65/60 and 75/100 (run time, min/%B). A Varian Star chromatographic system (50 mL pump head) was employed for purifications, using an elution system composed of A, 0.1% TFA in water, and B, 0.05%TFA in a mixture acetonitrile/water (4:1, v/v). Detection of eluting compounds was online monitored at 280 nm.

### Mass spectrometry analysis

Molecular mass measurement by MALDI-TOF: The sample was analysed by an Axima Confidence MALDI-TOF MS (Shimadzu, Japan) in positive linear mode on a 384-spot stainless-steel sample plate. Spots were coated with 1 µL 5 mg/mL CHCA previously dissolved in matrix diluent (Shimadzu, Japan), and the solvent was allowed to air dry. Then 0.5 µL sample or standard was applied onto the dry pre-coated well and immediately mixed with 0.5 µL matrix; the solvent was allowed to air dry. All spectra were acquired in the positive ion linear mode using a 337-nm N2 laser. Ions were accelerated from the source at 20 kV. A hundred profiles were acquired per sample, and 20 shots were accumulated per profile. The equipment was calibrated with a standard mixture provided in the TOFMixTM MALDI kit (Shimadzu, Japan). Measurements and MS data processing were controlled by the MALDI-MS Application Shimadzu Biotech Launchpad 2.9.8.1 (Shimadzu, Japan).

Sequencing by LC-MS/MS: The sample was reduced with 5 mM DTT for 20 min at RT, then carbamidomethylated with 50 mM iodoacetamide for 20 min at 37 °C, and subsequently digested with trypsin (ThermoFisher Scientific, 900,589), at a 1:50 ratio (enzyme: protein) for 16 h at 37 °C. A 15 µl aliquot of the digested sample was analyzed using an Orbitrap Elite Hybrid mass spectrometry system (Thermo Fisher Scientific) online coupled to an U3000 RSLCnano (Thermo Fisher Scientific) uPLC as described [40]. XCalibur 2.2 SP1.48 (Thermo Fisher Scientific, Bremen, Germany) was used for data-dependent tandem mass spectrometry (MS/MS) analyses. Database search (PEAKS DB) was performed using PEAKs X + studio [55]. For peptide identification, MS/MS spectra were correlated with the UniProt human reference proteome set (Uniprot release 2023_03; 20,423 reviewed entries). Parent mass error tolerance and fragment mass error tolerance were set at 15 ppm and 0.5 Da, respectively. Maximal number of missed cleavages was set at 3. Carbamidomethylated cysteine was considered as a fixed modification, and methionine oxidation as a variable modification. False discovery rates were set on the peptide level to 1%.

### Statistical analysis

All statistical analyses as mentioned in the figure legends were performed using GraphPad Prism v8.2.1 (GraphPad Software). Significance was calculated using non-parametric tests for paired samples (Wilcoxon-Rank test, paired t-test). Differences were considered significant when p-value < 0.05.

## Results

### Activity of lysozyme against extracellular *Mtb*

**Fig. 1:**
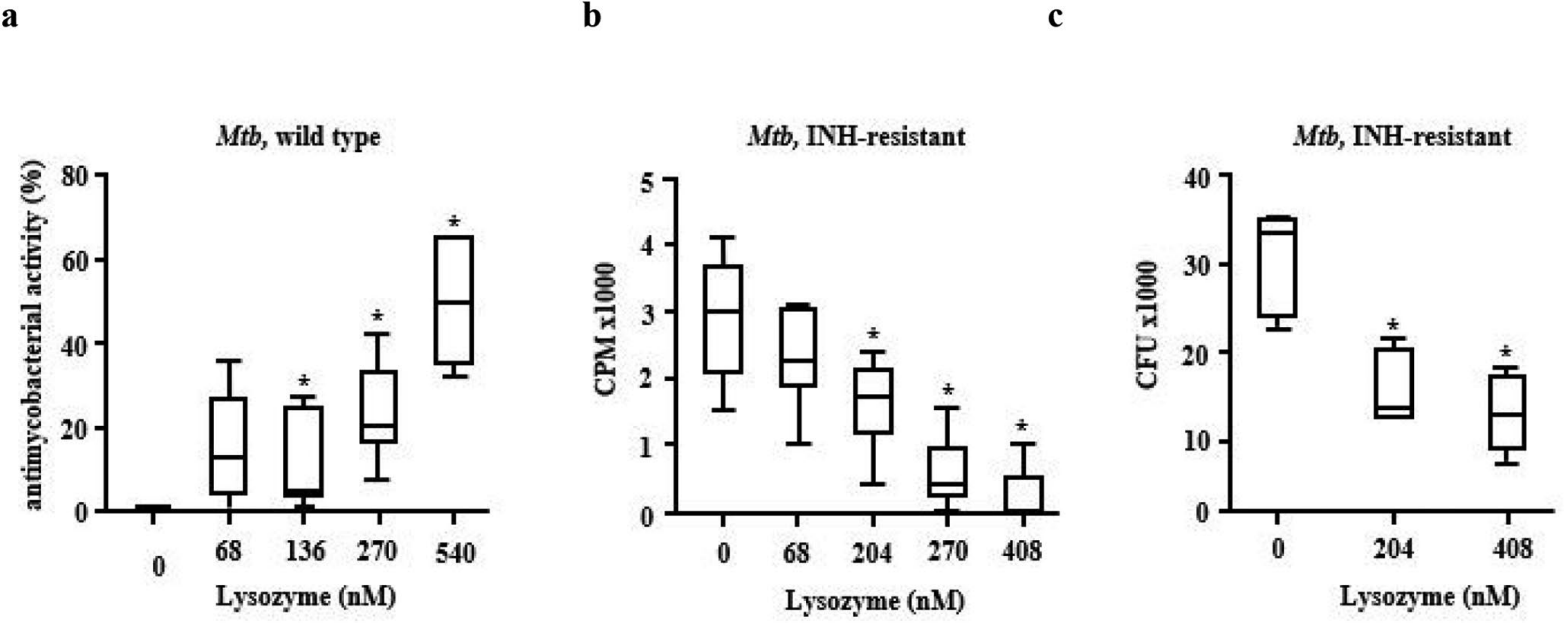
Activity of lysozyme against extracellular ***Mtb.*(a)** 2 × 10^6^ extracellular *Mtb* were incubated with increasing concentrations of lysozyme (stock concentration 0.1 mg/ml) for 3 days, followed by incubation with ^3^H-Uracil for 16 h. ^3^H-Uracil uptake was measured by scintillation counting. The antimycobacterial activity was calculated by comparison the counts per minute with the untreated control. Boxplots show the antimycobacterial activity (%) as median, upper and lower quartile as well as minimal and maximal value of n = six independent experiments. **(b)** 2 × 10^6^ extracellular INH-resistant *Mtb* were incubated with increasing concentrations of lysozyme for 3 days, followed by incubation with ^3^H-Uracil for 16 h. ^3^H-Uracil uptake was measured by scintillation counting. Boxplots show the counts per minute as median, upper and lower quartile as well as minimal and maximal value of n = six independent experiments **(c)** 2 × 10^6^ extracellular INH-resistant *Mtb* were incubated with increasing concentrations of lysozyme. After 3 days the number of viable bacilli was determined by plating samples in 10-fold dilutions on 7H11 agar plates and counting the colony forming units after 14d of culture. Boxplots show the count of colony forming units as median, upper and lower quartile as well as minimal and maximal value of n = six independent experiments. Statistical analyses of (a)-(c) were performed using non-parametric Wilcoxon-Mann test for unpaired samples and significance is indicated by an asterix

To investigate whether purified, human lysozyme inhibits the metabolism of extracellular *Mtb*, we compared the effect of increasing concentrations of lysozyme on the bacterial uptake of ^3^H-Uracil. Lysozyme showed a dose-dependent antimycobacterial activity against *Mtb* within the range of 68nM − 540nM (Fig. 1a). To ascertain that the reduced incorporation of uracil correlates with reduced viability of *Mtb*, lysozyme-treated bacilli were plated on Middlebrook agar plates. Lysozyme-exposure reduced the bacterial viability from approximately 10.000 CFU to 300 CFU (at 408nM, not shown). Lysozyme was equally efficient in inhibiting the growth of a strain resistant against the first line tuberculosis drug INH as determined by ^3^H-Uracil uptake (Fig. 1b) and determination of CFU (Fig. 1c).

### Activity of lysozyme against extracellular non-tuberculous mycobacteria

**Fig. 2:**
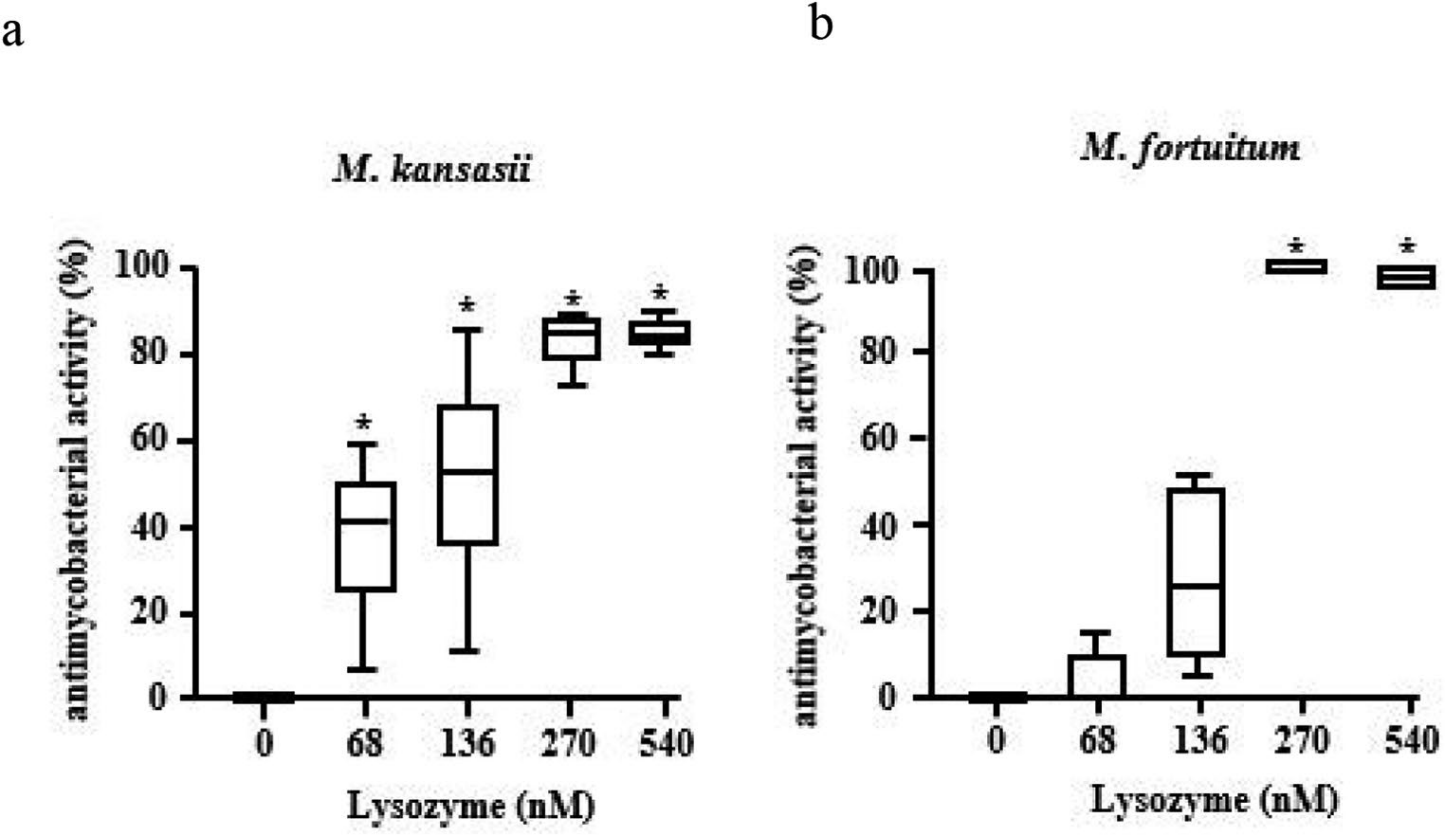
Activity of lysozyme against extracellular non-tuberculous mycobacteria. (**a**) 2 × 10^6^ extracellular *M. kansasii or* (**b**) *M fortuitum* were incubated with increasing concentrations of lysozyme (stock concentration 0.1 mg/ml) for 48 h, followed by incubation with ^3^H-Uracil for 16 h. ^3^H-Uracil uptake was measured by scintillation counting. The antimycobacterial activity was calculated by comparison the counts per minute with the untreated control. Boxplots show the antimycobacterial activity (%) as median, upper and lower quartile as well as minimal and maximal value of n = six independent experiments. Statistical analyses of (**a**)-(**b**) were performed using non-parametric Wilcoxon-Mann test for unpaired samples and significance is indicated by an asterix

Besides mycobacteria belonging to the *Mtb*-complex, non-tuberculous mycobacteria may cause severe infections particularly in immuno-suppressed individuals. To test the effect of lysozyme on non-tuberculous mycobacteria we selected *M. kansasii* as a representative slow growing- and *M. fortuitum* as a typical fast-growing strain, respectively. Based on the distinct cultural requirements we optimized the measurement of metabolic activity by reducing the exposure-time to lysozyme to 48 h (not shown). Lysozyme showed a dose-dependent antimycobacterial effect against *M. kansasii* reaching 84% activity at 540nM (Fig. 2a). The effect of lysozyme against the fast-growing strain *M. fortuitum* was even more striking resulting in sterile cultures at 270nM (Fig. 2b).

### Repetitive exposure to lysozyme does not induce *Mtb*-resistance

**Fig. 3:**
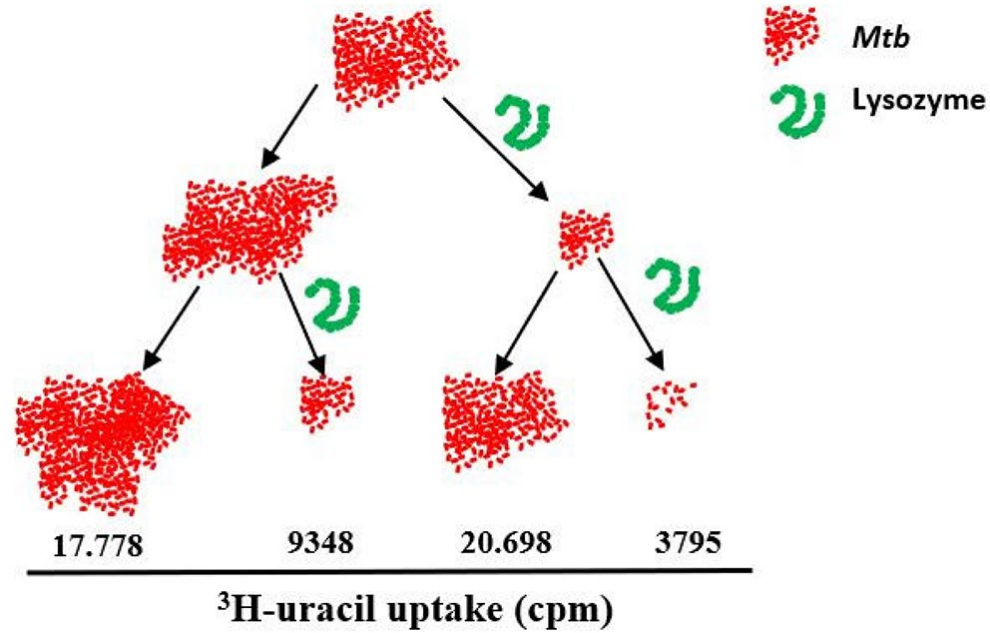
Repetitive exposure to lysozyme does not induce *Mtb*-resistance. *Mtb* (represented as red dots) was incubated in the absence or presence of lysozyme (408 nM, stock concentration 0.1 mg/ml). After 6 days the bacteria were washed intensively at high speed (3000 rpm) to remove lysozyme. Bacteria were then re-exposed (or not) to lysozyme for 3 days, followed by incubation with ^3^H-Uracil for 16 h to determine the metabolic activity of *Mtb*. All samples were performed in triplicates. The graph shows a representative result of five independent experiments

Selecting drug-resistant mutants during treatment is a major obstacle in controlling tuberculosis infection. Since lysozyme is a characteristic amphipathic and cationic AMP that acts by physical disruption of the cell membrane, we hypothesized that it would not favor the development of resistance in *Mtb* after prolonged and repetitive exposure. To address this hypothesis, we exposed *Mtb* to lysozyme twice (first for six, then for four days) and compared the uptake of ^3^H-Uracil to untreated or single-shot treated cultures (Fig. 3). The results in five independent experiments suggest that repetitive exposure of *Mtb* to lysozyme was even more efficient in inhibiting metabolic activity than the single exposure. The high variability in the absolute cpm-counts in the independent experiments precluded a statistically valid evaluation. This finding argues against the rapid selection of resistant mutants as described for single treatment with rifampin or streptomycin.

### Co-localization of endogenous lysozyme and *Mtb* in macrophages

**Fig. 4:**
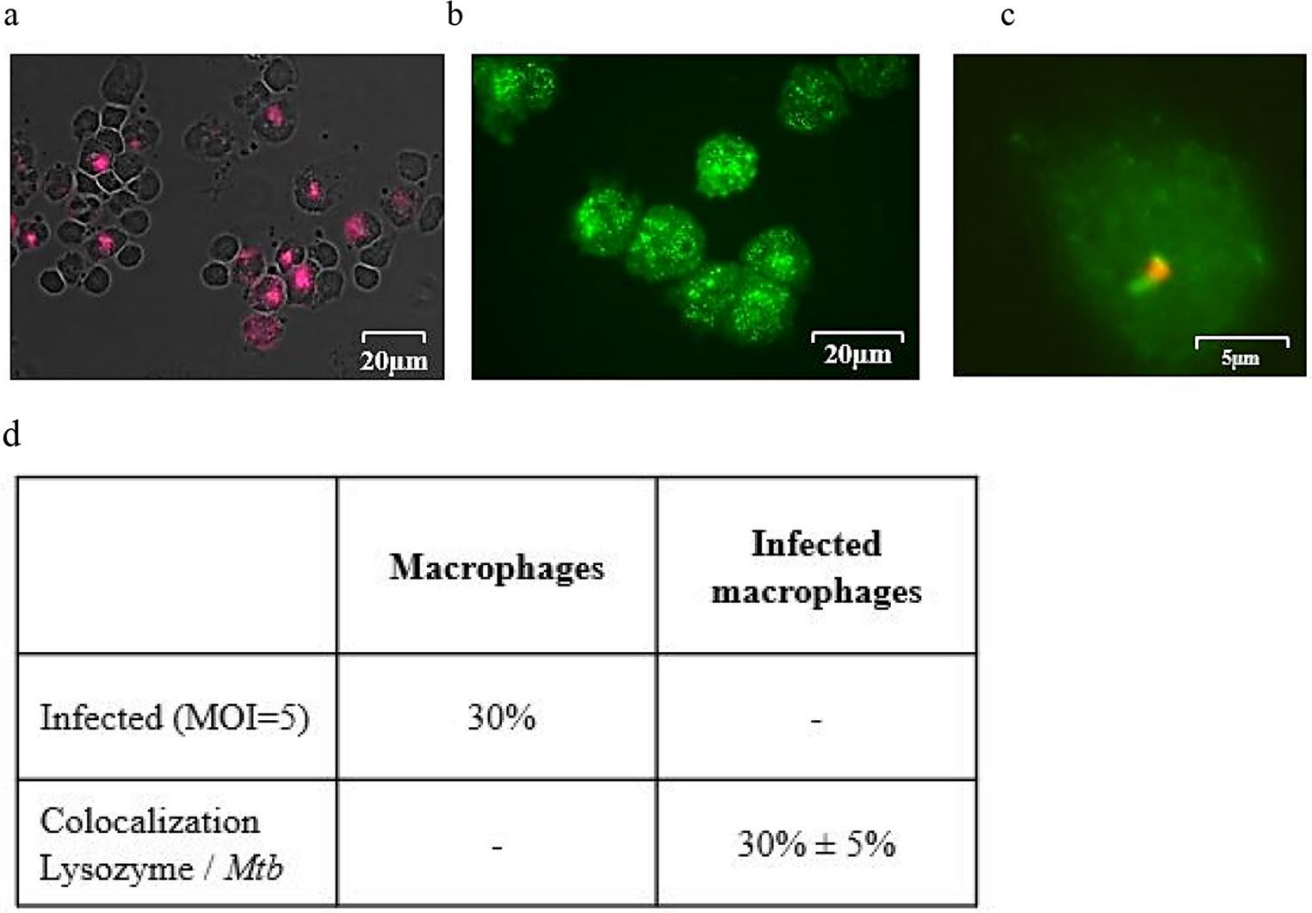
Expression of endogenous lysozyme and co-localization with *Mtb* in macrophages. **(a)** Lysozyme was labelled using an unconjugated monoclonal antibody and detected by an anti-rabbit-Alexa647 antibody in PBMCs as staining control. **(b)** Lysozyme was labelled using an unconjugated monoclonal antibody and detected by an anti-rabbit-Cy2 antibody in macrophages. **(c)** Macrophages were infected with *Mtb* (MOI = 5) in an 8-chamber slide. Lysozyme was labelled using an unconjugated monoclonal antibodyand detected by an anti-rabbit-Cy2 antibody. *Mtb* was labelled using a lipoarabinomannan-specific antibody and an anti-mouse-Cy3 antibody. Slides were analyzed under a fluorescence microscope. The panel demonstrates a representative section of a *Mtb*-infected macrophage with a co-localization of *Mtb* (red) and lysozyme (green), *n* = 5 experiments. **(d)** Quantification of (c)

In vivo, *Mtb* primarily resides intracellularly inside macrophages. Inside monocyte-derived macrophages- as used in our experiments- *Mtb* readily multiplies and destroys the host cell within 5–7 days in culture. Therefore, we hypothesized that our macrophages lack lysozyme and are, hence, permissive for mycobacterial overgrowth. However, lysozyme was highly expressed in all monocytes (Fig, 4a, staining control) and macrophages as demonstrated by the granular staining pattern in the immunofluorescence staining (Fig. 4b). Importantly, lysozyme also co-localized with *Mtb* in infected macrophages in 30% of all infected cells (Fig. 4c and d). By inference, the co-localization of endogenous lysozyme with *Mtb* in the mycobacterial compartment is not sufficient to prevent bacterial multiplication.

### Effect of lysozyme on the viability of intracellular *Mtb*

**Fig. 5:**
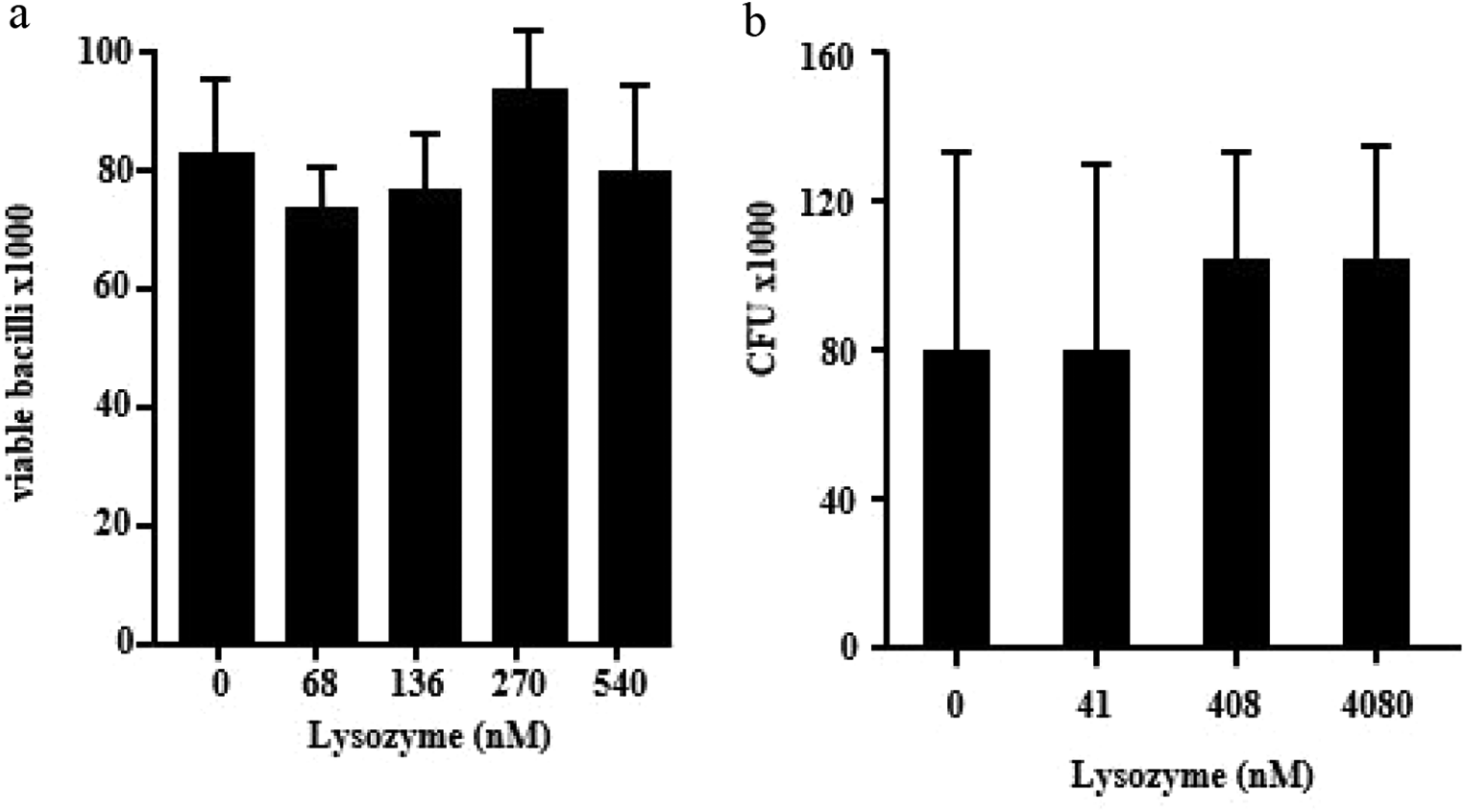
Effect of lysozyme on the viability of intracellular *Mtb*. **(a)** Macrophages were infected with *Mtb* (MOI = 5) and incubated with different concentrations of lysozyme (stock concentration 0.1 mg/ml). After 3d cells were lysed with distilled water and transferred into a MGIT-tube. The time to positivity was determined in a BacTec. The number of viable bacilli was determined by comparison of the time to positivity to a standard curve using defined numbers of extracellular *Mtb*. The graph shows the results (mean + SEM) obtained with macrophages of n = four independent donors and experiments. **(b)** Macrophages were infected with *Mtb* (MOI = 5) and incubated with different concentrations of lysozyme for 88 h. Afterwards, the bacteria were poured over the surface of a 7H11 Middlebrook agar plate. After 14 days of incubation, the colony forming units were counted. Bars show the mean of CFUs (+ SEM) as value of n = four independent donors and experiments

We speculated that application of exogenous lysozyme might be more efficient than endogenous lysozyme possibly by distinct trafficking or simply by increasing the bio-active concentration at the site of infection. To test this, we incubated *Mtb*-infected macrophages with exogenous lysozyme for three days and transferred cell lysates into MGIT tubes to perform a mycobacterial growth inhibition assay (MGIA). The time to positivity served as a correlate of mycobacterial load and was compared between the untreated and treated samples. However, lysozyme did not inhibit the mycobacterial growth in the MGIA (Fig. 5a). This inactivity was confirmed by conventional plating of cell lysates on Middlebrook agar and determining the number of CFU (Fig. 5b). Taken together, lysozyme is active against extracellular *Mtb*, but neither endogenous- nor exogenous lysozyme limit the multiplication of *Mtb* residing in human macrophages.

### Activity of Lys-H1 against extracellular *Mtb*

**Fig. 6:**
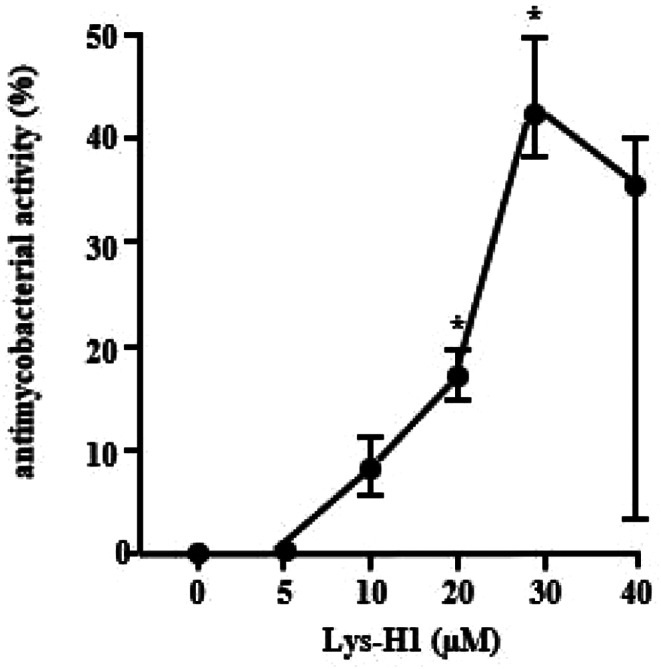
Activity of Lys-H1 against extracellular *Mtb.* 2 × 10^6^ extracellular *Mtb* were incubated with increasing concentrations of Lys H-1 (stock concentration 0.1 mg/ml) for 3 days, followed by incubation with ^3^H-Uracil for 16 h. ^3^H-Uracil uptake was measured by scintillation counting. The antibacterial activity was calculated by comparison the counts per minute with the un-treated control. All samples were performed in triplicates. The graph shows the results (mean + SEM) of n = seven independent experiments, significance is indicated by an asterix

One possibility for the inactivity of exogenous lysozyme against intracellular *Mtb* could be the degradation by proteases present in the culture medium or inside the macrophage. Therefore, we synthesized a smaller peptide of lysozyme (Lys-H1), which contains the putative active domain of lysozyme [20](AS 1–17). First, we tested whether Lys-H1 would maintain the antimicrobial activity of the parental protein against extracellular *Mtb*. Lys-H1 showed a dose-dependent reduction in the uptake of ^3^H-Uracil in the range between 10–30µM (Fig. 6). Therefore, the lysozyme-derived peptide Lys-H1 maintained the antimicrobial activity, albeit at higher molar concentrations. Thus, in comparison to lysozyme, Lys-H1 showed almost a 100-fold reduced activity.

### Uptake of Lys-H1 into macrophages

**Fig. 7:**
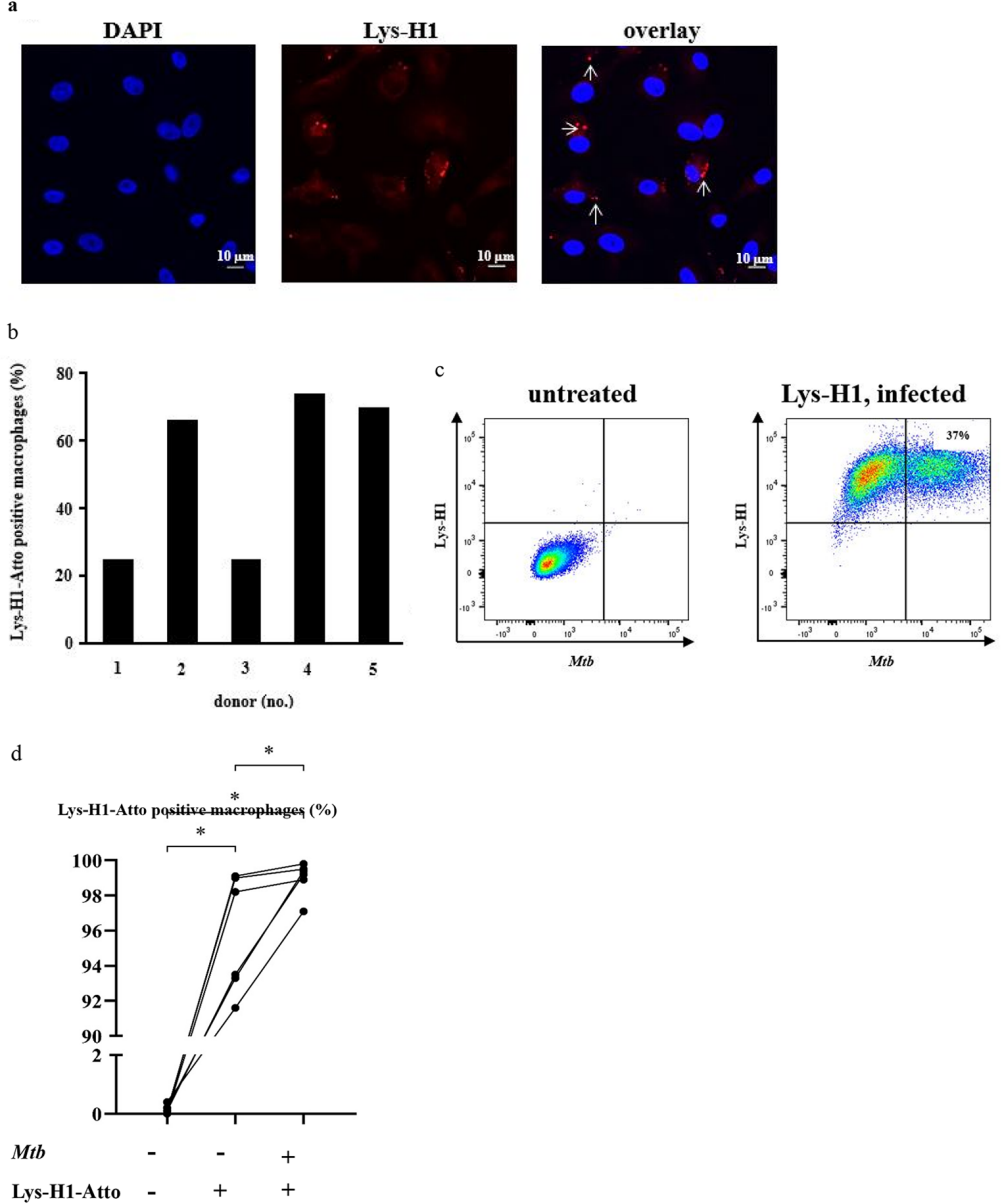
Uptake of Lys-H1 into macrophages. **(a)** 1 × 10^5^ macrophages were incubated with Lys-H1-Atto (15µM) for 24 h and analyzed by confocal laser microscopy. The panels show the staining of the nuclei (DAPI, blue), Lys-H1 (red) and the overlay. Agglomerations of Lys-H1-Atto are highlighted by arrows. The pictures show representative pictures of n = five independent donors and experiments. **(b)** Quantification of the uptake of Lys-H1-Atto in macrophages of all five donors (a). **(c)** 5 × 10^5^ macrophages were infected with succinimidyl-ester stained *Mtb* for 2 h (MOI = 12) and Lys-H1-Atto (50nM) was added for an additional 24 h. Samples were harvested and analyzed by flow cytometry. Results of one representative experiment are shown (*n* = 5). **(d)** Quantification of the uptake of Lys-H1-Atto in infected macrophages of all donors, significance is indicated by an asterix

To investigate the uptake and intracellular localization of LysH-1, we synthesized Lys-H1 linked to a fluorescent dye (Lys-H1-Atto, red). Lys-H1-Atto was incubated with macrophages and cells were analyzed by confocal laser microscopy. After 24 h of co-incubation the macrophages had taken up different amounts Lys-H1 as indicated by a diffuse punctate staining and larger agglomerates in a subset of macrophages (Fig. 7a, b). To strengthen and extend this phenotypic observation, we analyzed Lys-H1-Atto-treated macrophages, which had been infected with fluorochrome-labelled *Mtb* by flow cytometry. The flow cytometry studies showed that virtually all macrophages had taken up Lys-H1 (Fig. 7c, d). Consequently, all infected macrophages (24–47%) also stained positively for Lys-H1, providing the prerequisite for Lys-H1 to exert antimicrobial activity against intracellular *Mtb*.

### Effect of Lys-H1 on the viability of intracellular *Mtb*

**Fig. 8:**
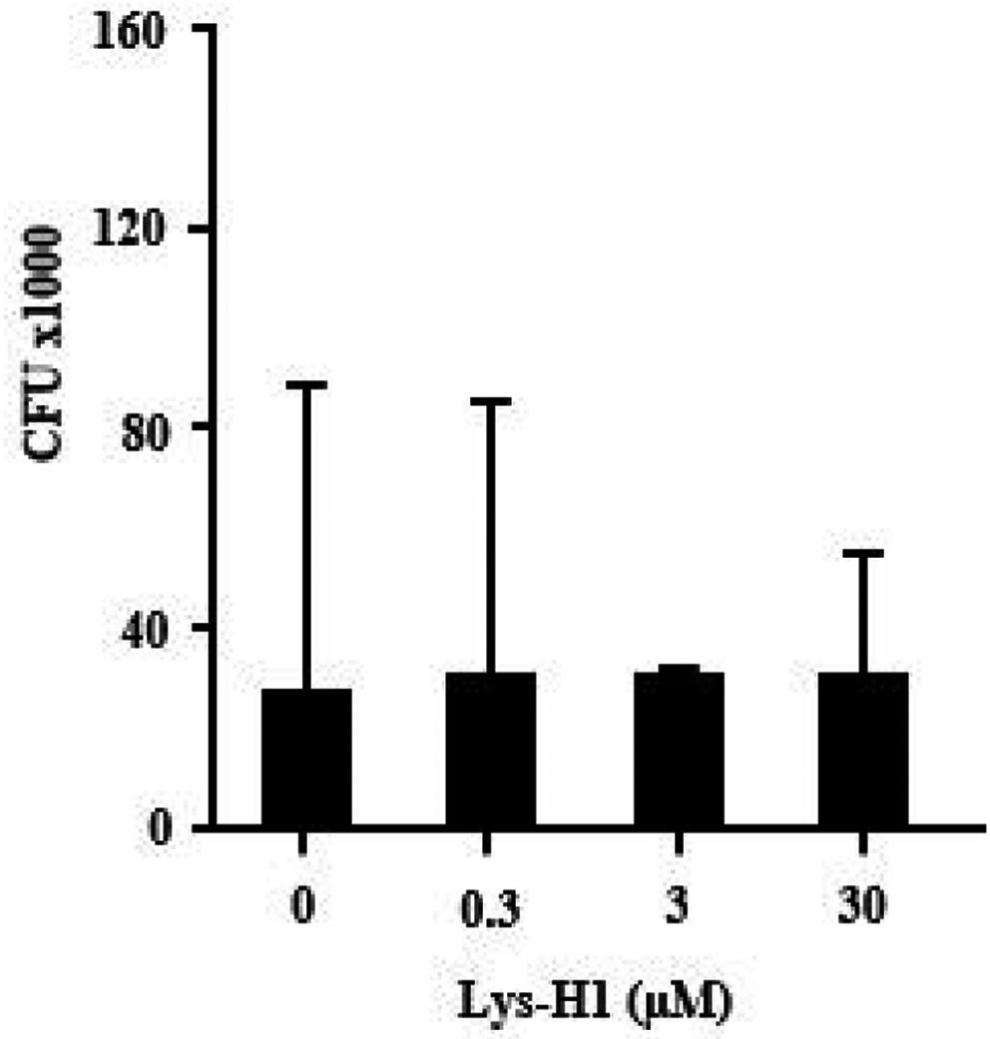
Effect of Lys-H1 on the viability of intracellular *Mtb*. Macrophages were infected with *Mtb* (MOI = 5) and incubated with different concentrations of Lys-H1 (stock concentration 0.1 mg/ml) for 88 h (similar to measurement of metabolic activity, 72 h before adding uracil and 18 h in the presence of uracil). Afterwards, the bacteria were poured over the surface of a 7H11 Middlebrook agar plate. After 14 days of incubation, the colony forming units were counted. Bars show the mean of CFUs (+ SEM) as value of n = three independent experiments

Since Lys-H1 enters *Mtb*-infected macrophages, we next evaluated whether this triggers a reduction in the viability of *Mtb* by determining the number of CFU in untreated and Lys-H1-treated macrophages (Fig. 8). Lys-H1 had no effect on the number of CFU in the range between 0.3 and 30 µM. Taken together, lysozyme and its smaller derivative Lys-H1 show potent antimicrobial activity against extracellular mycobacteria. Both efficiently enter *Mtb*-infected macrophages but fail to affect the viability of the intracellular pathogen.

### Lysozyme is a prominent antimycobacterial protein in human haemofiltrates

**Fig. 9:**
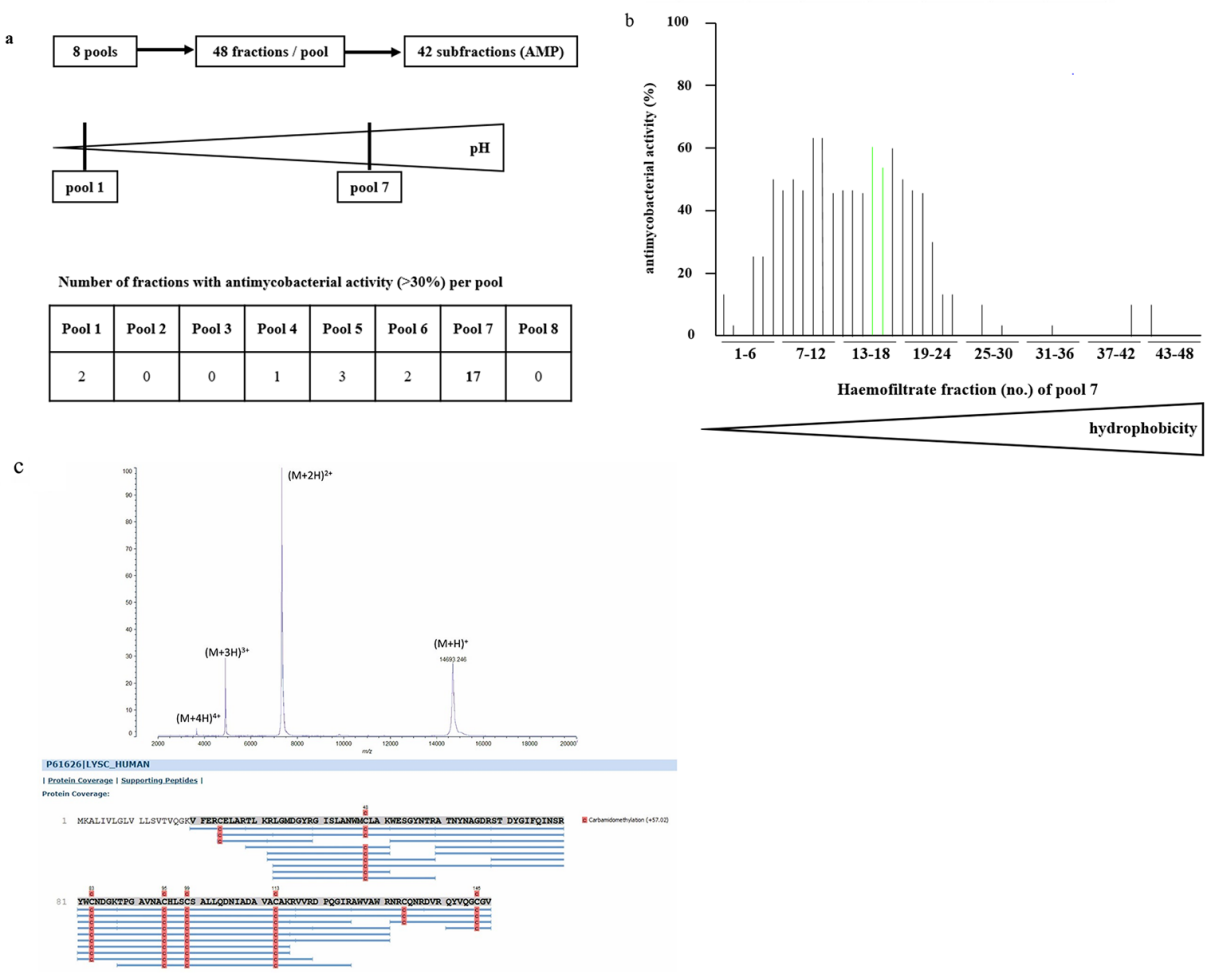
Lysozyme is a prominent antimycobacterial compound in human haemofiltrates. **(a)** Overview of all tested pools and fractions. **(b)** Haemofiltrate fractions (10 µl) were incubated with extracellular *Mtb* for 3 days, followed by incubation with ^3^H-Uracil for 16 h. ^3^H-Uracil uptake was measured by scintillation counting. The antimicrobial activity (%) was calculated by comparison the counts per minute with the untreated control. **(c)** Active fractions were analyzed by mass spectrometry. * indicates the identified proteins. A.u. = arbitrary unit. *m/z* = mass to charge ratio

To estimate the potential and significance of lysozyme in contributing to early defense against *Mtb*-infection we analyzed a peptide library obtained from pooled haemofiltrate of a large cohort of dialysis patients for the presence of AMPs with activity against *Mtb*. The starting point were 8 peptide pools generated according to pH, charge and hydrophobicity [43]. Every pool consists 48 fractions. Aliquots of the fractions were co-incubated with extracellular *Mtb*, and the uptake of ^3^H-Uracil was determined after three days of incubation (Fig. 9a). Peptide pools with antimycobacterial activity (arbitrarily defined as > 30% reduction of cpm) were further sub-fractionated and re-evaluated for antibacterial activity (not shown). MALDI-TOF analysis of one active chromatographic fraction obtained after two rounds of screening, showed several high-intensity signals originated from only one molecular species (Fig. 9b), which was identified as human lysozyme (Uniprot P61626) by MS/MS sequencing (Fig. 9c). This approach demonstrates that lysozyme is one of several dominant AMP in human serum which can be purified amongst hundreds of thousands of peptides by screening for antimicrobial activity against the highly virulent human pathogen *Mtb*.

## Discussion

Since the days of Alexander Fleming lysozyme has been recognized as an important component of protection early after infection with gram-positive bacteria. Here we demonstrate that purified human lysozyme is also active against the major human pathogen *Mtb*, including drug-resistant strains. Despite intracellular co-localization with *Mtb*, lysozyme does not inhibit the growth of the bacteria residing in human macrophages. Lysozyme was also identified in an unbiased screen for antimycobacterial activity of a peptide library of human serum. Recently, we identified two additional peptides with antimycobacterial activity against Mtb -LL-37 and Angiogenin- from human peptide libraries further highlighting the power of this strategy to identify endogenous antimicrobial peptides.

Based on these findings we hypothesize, that lysozyme contributes to protection against early-stage tuberculosis. It may inhibit extracellular *Mtb* depending on the individual levels in human serum as well as intracellular *Mtb* through colocalization with the bacteria in macrophages.

Lysozyme showed potent activity against extracellular *Mtb* in the nanomolar range and co-localized with *Mtb* in human macrophages. Therefore, it was unexpected, that exogenous lysozyme failed to inhibit the growth of intracellular *Mtb*. There are several explanations for this discrepancy.

First, exogenous lysozyme may not be taken up in sufficient levels into macrophages, leading to concentrations not high enough to effectively inhibit antimycobacterial growth. Further experiments measuring intracellular levels of lysozyme and investigating different transport mechanisms of lysozyme into macrophages may answer this question. Furthermore, *Mtb* has evolved mechanisms to escape the action of lysozymes which may be activated inside the host cell. Normally, lysozyme hydrolyzes the β-1,4-glycosidic bonds between N-acetylglucosamine and N-acetylmuramic acid of the peptidoglycan of the bacterial cell wall, thereby inducing cell lysis [7, 9, 49]. These targets are also components of the mycobacterial cell wall. However, *Mtb* owns a special gene encoding the protein Rv1096, which leads to the deacetylation of glucosamine and inhibits this effect of lysozyme [52]. In addition, virulent *Mtb* strains express the surface lipoprotein LprI, which counteract the antimicrobial effect of lysozyme [44]. The gene underlying the LprI protein is transcribed together with another gene (glbN), which is upregulated after macrophage infection. Similarly, LprI expression also increases when *Mtb* enters its host cell. However, since particularly extracellular mycobacteria were studied in this work, that could be a reason for the lack of protection of LprI in these extracellular bacteria. On the other hand, the upregulation of LprI in intracellular *Mtb* may be a reason for the lack of activity of lysozyme against intracellular *Mtb*. Furthermore, the expression of LprI depends on the growth phase of mycobacteria. It is low in the exponential phase and increases to its maximum in the stationary phase [44]. The use of mycobacteria that may have been in their exponential growth phase could be another reason for the lack of lysozyme resistance in *Mtb*.

Secondly, the function of lysozyme could be modulated by proteases inside the cytosol or the phagolysosome of infected macrophages. Lysosomal aspartic proteases of azurophil granules including cathepsin D and pepsin have been shown to affect the function of lysozyme [19, 48]. The inactivity of Lys-H1, a small derivative of lysozyme (helix-1, AS 1–17) against intracellular *Mtb* (Fig. 8) argues against this hypothesis. We selected Lys-H1 based on its superior activity against Gram-negative and Gram-positive bacteria as compared to the parental molecule and other synthetic peptides derived from lysozyme [20]. The lysozyme-derived peptides were significantly more active against Gram-positive and Gram-negative bacteria than the parental molecule. In contrast, the activity of Lys-H1 was approx. 100-fold less against virulent *Mtb* than lysozyme, reflecting the uniqueness of the mycobacterial cell wall. We speculate that the charge (+ 3) and the isoelectric point (10.05) of Lys-H1 are disadvantageous for the penetration of the lipid-rich mycobacterial cell wall. Furthermore, the shortening of lysozyme to Lys-H1 may affect and limit its antimycobacterial activity. The uptake and trafficking of lysozyme or its derivatives could be improved by engineering delivery vehicles that specifically target macrophages [12] and support the migration of effector molecules into the mycobacterial compartment. The efficacy of this approach has been recently demonstrated for the endogenous antimycobacterial peptides gran1 [33], Angiogenin [32] and Napsin [3] which were integrated into liposomes or mesoporous nanoparticles. These delivery vehicles will also protect the bioactive compounds from proteolytic degradation and offer the intriguing possibility to include immune modulatory molecules with adjuvant activity [28].

Finally, lysozyme has multiple and diverse effects on immune cells independently of the antimicrobial activity [38]. One mechanism is to trigger the activation of macrophages indirectly as a result of lysozyme-mediated bacterial degradation. This will lead to the release of pathogen-associated molecular patterns (PAMPs) that induce proinflammatory responses for example by activating the inflammasome [45]. Additionally, lysozyme can directly modulate serum complement activation resulting in the inhibition of macrophage recruitment and function [34]. Interestingly, Lys-H1 is a potent inhibitor of the lipopolysaccharide-stimulated release of TNF-a, IL-6 and IL-1ß [21] in murine macrophages. Since complement and cytokines are critical for supporting the elimination of *Mtb* by macrophages [14] the immune modulatory functions of lysozyme could counteract the antimicrobial activity of lysozyme and explain its inactivity against intracellular bacteria.

The possible role of lysozyme in protection against tuberculosis was first indicated more than 40 years ago using egg white lysozyme [25]. Based on this pioneering study lysozyme was detected in high levels in BCG-vaccinated rabbits [29], alveolar macrophages [30], granulomas of mycobacteria-infected rabbits [37] and in serum and bronchoalveolar lavage of tuberculosis patients [31, 36]. However, treatment of *Mtb*-infected mice with lysozyme showed no beneficial effect [50]. The most likely source for lysozyme in vivo are neutrophils because lysozyme was identified as active component of azurophilic neutrophil granules [22]. The same study showed that lysozyme co-localizes with *Mycobacterium smegmatis* in macrophage cell lines and inhibits the intracellular multiplication of this avirulent mycobacterial species [22]. Here, we demonstrate that lysozyme co-localizes with virulent *Mtb* in primary human macrophages. Despite intracellular co-localization viability of the bacilli was not affected. This is in striking contrast to the potent antimicrobial activity of lysozyme against extracellular *Mtb* in the nanomolar range. This evidence reinforces the role of Lysozyme as a potential candidate for AMP-based antimycobacterial therapy.

## Data Availability

No datasets were generated or analysed during the current study.
